# Transcriptional Response to Tick-Borne Flavivirus Infection in Neurons, Astrocytes and Microglia In Vivo and In Vitro

**DOI:** 10.3390/v16081327

**Published:** 2024-08-19

**Authors:** Ebba Rosendal, Richard Lindqvist, Nunya Chotiwan, Johan Henriksson, Anna K. Överby

**Affiliations:** 1 Department of Clinical Microbiology, Umeå University, 90185 Umeå, Sweden; 2 The Laboratory for Molecular Infection Medicine Sweden (MIMS), Umeå University, 90187 Umeå, Sweden; 3 Chakri Naruebodindra Medical Institute, Faculty of Medicine Ramathibodi Hospital, Mahidol University, Samut Prakarn 10540, Thailand; 4 Department of Molecular Biology, Icelab, Umeå Centre for Microbial Research (UCMR), Umeå University, 90187 Umeå, Sweden

**Keywords:** tick-borne encephalitis virus, Langat virus, RNA sequencing, snRNA-seq, neuroinflammation, interferon signaling

## Abstract

Tick-borne encephalitis virus (TBEV) is a neurotropic member of the genus *Orthoflavivirus* (former *Flavivirus*) and is of significant health concern in Europe and Asia. TBEV pathogenesis may occur directly via virus-induced damage to neurons or through immunopathology due to excessive inflammation. While primary cells isolated from the host can be used to study the immune response to TBEV, it is still unclear how well these reflect the immune response elicited in vivo. Here, we compared the transcriptional response to TBEV and the less pathogenic tick-borne flavivirus, Langat virus (LGTV), in primary monocultures of neurons, astrocytes and microglia in vitro, with the transcriptional response in vivo captured by single-nuclei RNA sequencing (snRNA-seq) of a whole mouse cortex. We detected similar transcriptional changes induced by both LGTV and TBEV infection in vitro, with the lower response to LGTV likely resulting from slower viral kinetics. Gene set enrichment analysis showed a stronger transcriptional response in vivo than in vitro for astrocytes and microglia, with a limited overlap mainly dominated by interferon signaling. Together, this adds to our understanding of neurotropic flavivirus pathogenesis and the strengths and limitations of available model systems.

## 1. Introduction

Tick-borne encephalitis virus (TBEV) belongs to the family of *Flaviviridae* and the genus *Orthoflavivirus* (former *Flavivirus*), which contain several medically important pathogens such as West Nile virus (WNV), Japanese Encephalitis virus (JEV), and Zika virus (ZIKV). These flaviviruses are neuroinvasive and neurotropic, meaning they can invade and replicate within the central nervous system (CNS). TBEV can be transmitted to humans through the bite of an infected tick or through the consumption of unpasteurized dairy products [1,2]. TBEV infections are estimated to result in 10,000–12,000 hospitalizations annually [3], and 30–50% of these patients experience long-lasting neurological sequelae [4]. Although two safe and efficient vaccines are approved for use in Europe [5], the number of TBE cases continues to rise in several European countries [6]. This is likely a result of environmental factors allowing the virus to spread to new endemic areas, with low vaccination coverage and awareness amongst the population. Currently, no specific antiviral treatment against TBEV is approved for clinical use in Europe. Treatment is aimed at relieving disease symptoms, and patients rely largely on their immune systems to fight the infection.

Pathogenicity during TBEV infection is believed to be the result of both direct damage to neurons by the virus infection and immunopathology due to excessive inflammation. A better understanding of the virus-induced response during TBEV infections is, therefore, warranted and could enable the rational design of immunomodulatory therapeutics to limit disease consequences. Clinically, the immune response during TBEV infections has been studied by measuring the presence of various cytokines in serum and cerebrospinal fluid (CSF) [7] and analyzing the adaptive immune response from peripheral blood or CSF [8]. Alternatively, various animal models have been used to study TBEV infection [9,10,11]. In these models, as well as in human autopsy samples [12], TBEV has demonstrated a preference for infecting neurons. Histological analysis has shown inflammatory changes such as perivascular infiltrates, gliosis and neuronal damage within the CNS [10,11]. Additionally, changes in cytokine and chemokine patterns in CNS have been explored on both transcriptional and protein levels in animal models [13,14]. Until recently, these approaches have been limited to the analysis of average expression levels, presenting a global view of the complex environment in vivo, without being able to separate the relative contribution of specific cell types. For more detailed and mechanistic studies on specific cell types’ contribution to neuropathogenesis, CNS-derived cell lines [14,15] or primary cells of human or murine origin [16,17,18,19] have, therefore, been used. We recently explored the transcriptional response to Langat virus (LGTV) infection in whole mouse cortex by single-nuclei RNA sequencing (snRNA-seq) [20]. LGTV is a naturally low-virulent tick-borne flavivirus and a commonly used BSL-2 model virus for TBEV. An advantage of single-cell technologies is the possibility of capturing the cell-type-specific response to infection within a complex tissue, such as the brain. To what extent cellular crosstalk between cell types shapes the immune response to infection remains poorly characterized.

In this study, we compared the response of primary cells isolated from the cortex and infected in vitro with the response elicited by the corresponding cell type in vivo. To better correlate our findings to previous studies of TBEV, we also compared the transcriptional response to LGTV and TBEV infection in primary cells and can show a very similar but stronger immune response to TBEV infection. 

## 2. Materials and Methods

### 2.1. Viruses

Virus stocks of LGTV strain TP21, a kind gift from G. Dobler (Bundeswehr Institute of Microbiology, Munich, Germany), and recombinant TBEV strain Torö European subtype [21], passage number 3, were produced in VeroB4 cells, a kind gift from G. Dobler (Bundeswehr Institute of Microbiology, Munich, Germany). Supernatants were harvested on day 3–4 post-infection when cytopathic effects were apparent and titrated on VeroB4 cells using a focus-forming assay, as previously described [22].

### 2.2. Infection of Primary Cells

Cortical astrocytes and neurons were generated as previously described [16,23]. In brief, astrocytes were isolated from neonatal mice between postnatal days 1 and 4. Cerebral cortices were isolated in HBSS (Gibco, Waltham, MA, USA) containing 0.5% penicillin and streptomycin (Gibco, Waltham, MA, USA) and 0.4% glucose. Tissues were dissociated by trituration and seeded in poly-d-lysine-coated T-75 tissue culture flasks. Astrocytes were grown in Dulbecco’s modified Eagle’s medium (DMEM) supplemented with 10% heat-inactivated FBS (Thermo Fisher Scientific, Waltham, MA, USA), 0.2% penicillin and streptomycin (Gibco, Waltham, MA, USA) and 2 mM L-glutamine (Gibco, Waltham, MA, USA), with a purity of >80%. Primary cortical neurons were isolated from the cerebral cortices on embryonic day 17 and seeded onto poly-d-lysine-coated wells. Neurons were grown in neurobasal plus medium (Gibco, Waltham, MA, USA) supplemented with B27 plus supplement (Gibco, Waltham, MA, USA), 0.2% penicillin and streptomycin and allowed to differentiate for 7 days before infection; final cultures had a purity of >95%. Microglia were isolated from the astrocyte cultures as previously described [24]. In brief, confluent monolayers of mixed glial cells were subjected to solution containing 1:3 (0.25% trypsin: DMEM-F12) for 30 min at 37 °C and 5% CO_2_ in order to remove astrocytes from the mixed glial cells. The remaining cells were grown in (DMEM) supplemented with 10% heat-inactivated FBS (Thermo Fisher Scientific, Waltham, MA, USA), 0.2% penicillin and streptomycin (Gibco, Waltham, MA, USA) and 2 mM L-glutamine (Gibco, Waltham, MA, USA) and conditioned astrocytes medium (1:1); final cultures had a purity of >85%. Monolayers of neurons (MOI 0.01), astrocytes (MOI 0.1) and microglia (MOI 1) were infected with LGTV or TBEV for 1 h at 37 °C and 5% CO_2_ before the inoculum was removed and replaced with fresh medium. Cell supernatant was harvested at the indicated time points, and viral titers were determined by focus-forming assay.

### 2.3. Immunofluorescence Assay

Primary cells were grown in 8-well chambers (Sarstedt, Nümbrecht, Germany) and fixed (4% formaldehyde, PBS). Cells were permeabilized (PBS, 20 mM glycine, 0.5% Triton-X100) for 10 min at RT, blocked (PBS, 10% FBS) for 1 h, immunolabelled with primary antibodies (chicken anti-non-structural protein 5 (NS5); 1:1000 [20], rabbit anti-tubulin beta 3 class III (TUBB3); 1:2000; Biolegend, San Diego, CA, USA; Poly18020, rabbit anti-glial fibrillary acidic protein (GFAP); 1:500; Dako/Agilent Technologies, Santa Clara, CA, USA; Z0334, rabbit anti-ionized calcium-binding adapter molecule 1 (Iba1); 1:500; Histolab, Askim, Sweden; BC-CP290B) diluted in blocking buffer for 1 h and labeled with fluorescent secondary antibody (donkey anti-Rabbit IgG Alexa Fluor™ 488; 1:500; Invitrogen, Waltham, MA, USA; A-21206, goat anti-chicken IgY Alexa Fluor™ 555; 1:500; Invitrogen, Waltham, MA, USA; A-21206) for 1 h. Cells were imaged on a Leica SP8 confocal microscope, and image processing was performed using ImageJ (NIH).

### 2.4. RNA Extraction and RT-qPCR

LGTV-infected, TBEV-infected or mock-treated monolayers of neurons (300,000 cells), astrocytes (300,000 cells) and microglia (400,000 cells) were washed once with PBS and lysed in RA1 lysis buffer (Macherey-Nagel, Düren, Germany) at indicated time points. RNA was extracted using Nucleospin RNA plus mini kit (Macherey-Nagel, Düren, Germany) according to the manufacturer’s instructions. Then, 200 ng of RNA was used as input for cDNA synthesis (High-capacity cDNA Reverse Transcription kit, Thermo Fisher Scientific, Waltham, MA, USA). Viral RNA was quantified with qPCRBIO probe mix Hi-ROX (PCR Biosystems, London, UK) and primers recognizing LGTV NS3 (Forward primer: CATTGACAAGGCATGTGACAAGA, reverse primer: ACAGCACCATTCGGGTATGACT, probe: FAM-AGAGACAGATCCCTGATGG-BHQ) [25] or TBEV 3’ UTR (Forward primer: GGGCGGTTCTTGTTCTCC, reverse primer: ACACATCACCTCCTTGTCAGACT, probe: FAM-TGAGCCACCATCACCCAGACACA-BHQ) [26]. GAPDH and Rps18 were detected by QuantiTect primer assay (GAPDH; QT01658692, Rps18; QT02448075, Ifnb1; QT00249662, Rsad2; QT00109431 Qiagen, Venlo, Netherlands) and the qPCRBIO SyGreen mix Hi-ROX (PCR Biosystems, London, UK). All experiments were run on a StepOnePlus real-time PCR system (Applied Biosystems, Waltham, MA, USA).

### 2.5. Single-Nuclei RNA Sequencing and Pseudobulk Analysis

Pre-annotated snRNAseq datasets from the cerebral cortex of C57BL/6 WT mice were used [20]. Sample preparation and data processing are described in full in the original publication. In brief, 7- to 13-week-old mice were inoculated intracranially with 10^4^ PFU of LGTV, PBS or left untreated. Animals were euthanized by oxygen deprivation on day 5 post-infection when reaching the criteria for the humane endpoint. Cerebral cortexes of the hemisphere that did not receive needle injection from one male and one female mouse were pooled, and single-nuclei suspensions were generated by partial lysis of cells, FACS sorted and subjected to droplet-based single-cell RNA sequencing (10× Genomics, Pleasanton, CS, USA). Data for each condition (infected or uninfected) were given a simplified cell-type annotation and separated into biological duplicates based on the expression of sex-specific genes (*Xist* and *Eif2s3y*). Pseudobulk counts were generated by AggregateExpression, Seurat package version 4.1.0 [27], for each cell type and biological replicate separately. 

### 2.6. Bulk RNA Sequencing

Total RNA from infected or mock-treated monocultures (*n* = 2 per condition) was extracted using Nucleospin RNA plus mini kit (Macherey-Nagel, Düren, Germany) according to the manufacturer’s instructions. The Smart-Seq2 protocol was used to produce fragmented cDNA with Nextera sequencing adapters [28], and the cDNA was sequenced with Illumina Nextseq 550 with 37 bp PE (75 cycles kit). The reads were aligned using STAR 2.7.9a [29] to a custom reference genome, consisting of *Mus musculus* genome assembly (GRCm38), TBEV (Torö-2003 GenBank Accession no. DQ401140.3) and LGTV (Langat virus TP21, NCBI Reference Sequence: NC_003690). FeatureCounts v.2.0.1 was used to generate a count table.

### 2.7. Differentially Expressed Genes and Gene Set Enrichment Analysis

Data analysis was performed in R, version 4.2. Genes with a total count <10 were considered low-count genes and removed. Pairwise comparisons between the conditions were performed using DESeq2 version 1.36.0 [30] and normalized counts calculated by estimateSizeFactors. For analysis of differentially expressed genes, shrunken log2 fold changes (LFC) were calculated using lfcShrink and the shrinkage estimator ashr [31]. The cutoff for significance was set at shrunken log2 fold change > |1| and adjusted *p*-value < 0.05. Enrichment/over-representation analysis was performed using enrichR package version 3.1 on the Hallmark Gene Set curated gene lists [32,33] using significant DEGs, and gene set enrichment analysis was performed using fgsea package version 1.22.0 on the Reactome pathway curated gene set [34,35], ranking genes based on LFC without any LFC or *p*-value cutoff.

## 3. Results

### 3.1. Characterization of Primary Neurons, Astrocytes and Microglia

**Figure 1:**
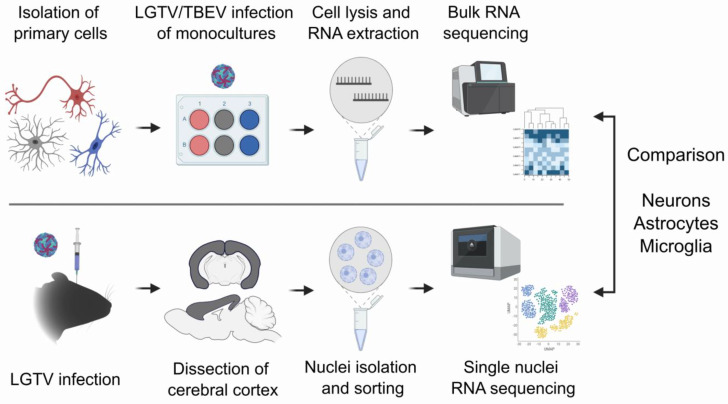
In vitro and in vivo models to study the transcriptional response to tick-borne flavivirus infection. In vitro monoculture model (top), primary murine neurons, astrocytes and microglia were isolated from the neonatal mice and grown as monocultures. Cells were infected with LGTV or TBEV and subjected to bulkRNA-seq at 24 h post-infection. In vivo model (bottom), snRNA-seq data of mouse cerebral cortex from LGTV-infected or uninfected animals at humane endpoint [20], analyzed as “pseudobulk”. Illustration created with BioRender.com.

Primary cultures of CNS-derived cells are a well-established model system for mechanistic studies of neurotropic viruses. However, primary cells are often derived from neonatal/very young mice and grown as monocultures in vitro and are, thus, not exposed to signals from other cell types. These conditions may affect the cellular response and transcriptional outcome after infection, making the translation from in vitro to in vivo difficult. To shed light on the similarities and differences in cellular response in the different models, we set out to investigate the transcriptional response to flavivirus infection in vitro and in vivo (Figure 1). For the in vitro model, we isolated primary neurons and glial cells from the cerebral cortex of C57BL/6 mice. Glial cells were separated into astrocytes and microglia, whereas neurons were differentiated in vitro. We infected these monocultures with LGTV or TBEV for 24 h and subjected them to total RNA isolation and bulk RNA sequencing (bulkRNA-seq). To compare with transcriptional response in vivo, we used data from the snRNA-seq performed in a previous study by Chotiwan et al., 2023 [20]. This consists of data from the cerebral cortex of LGTV-infected or uninfected mice from the same genetic background, which we analyzed as “pseudobulk”, i.e., averaged the gene expression of all cells in a group to simulate a bulk transcriptomics analysis.

**Figure 2:**
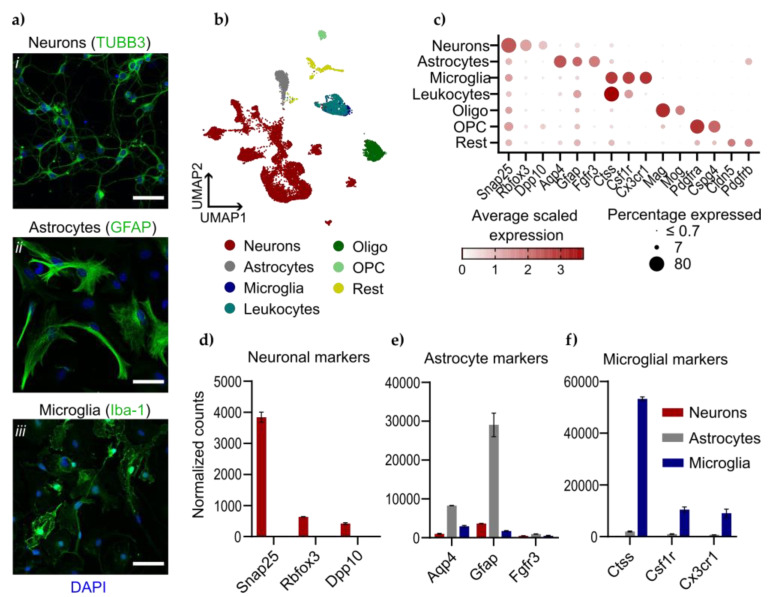
Characterization of neurons, astrocytes and microglia from in vitro and in vivo models. (**a**) Monocultures of primary cells were fixed, permeabilized and immunolabeled against (i) TUBB3 (neurons), (ii) GFAP (astrocytes) or (iii) Iba1 (microglia) together with nuclear staining with DAPI. Scale bar 50 µm. (**b**) 14,196 single nuclei from the cerebral cortex of uninfected and LGTV-infected WT C57BL6 mice (in vivo model), colored by assigned identities and visualized by Uniform Manifold Approximation and Projection (UMAP). The annotation Rest included small cell populations, such as vascular leptomeningeal cells (VLMCs), pericytes, endothelial cells and choroid plexus epithelial cells. (**c**) Dot plot showing expression of marker genes used to identify major cell types in (**c**), color represents average scaled expression in group and size represents the percentage of cells expressing the marker. Expression of cell-type-specific marker genes for neurons (**d**), astrocytes (**e**) and microglia (**f**) in primary monocultures measured by bulkRNA-seq, presented as normalized counts.

The cellular identities of the in vitro monocultures were confirmed by immunofluorescent staining against cellular markers TUBB3; neurons, GFAP; astrocytes or Iba-1; microglia (Figure 2a). SnRNA-seq of mouse cortex identified distinct cell populations corresponding to the main cell types found in vivo, such as neurons, astrocytes, microglia and oligodendrocytes/oligodendrocyte progenitor cells (OPCs) (Figure 2b). To evaluate how well the primary cells resemble the cell populations in vivo, we examined the expression of cell-type-specific marker genes identified in the snRNA-seq dataset (Figure 2c) in the primary cell cultures using bulkRNA-seq (Figure 2d–f). We found that the expression profile of the primary cultures was dominated by the expected marker genes, and only one marker gene, the astrocyte marker *Fgfr3*, was not enriched in the primary cells. Together, this verifies the cellular identities of our primary monocultures.

### 3.2. LGTV Infection Induces a Similar But Weaker Immune Response Than TBEV in Primary Cells

**Figure 3:**
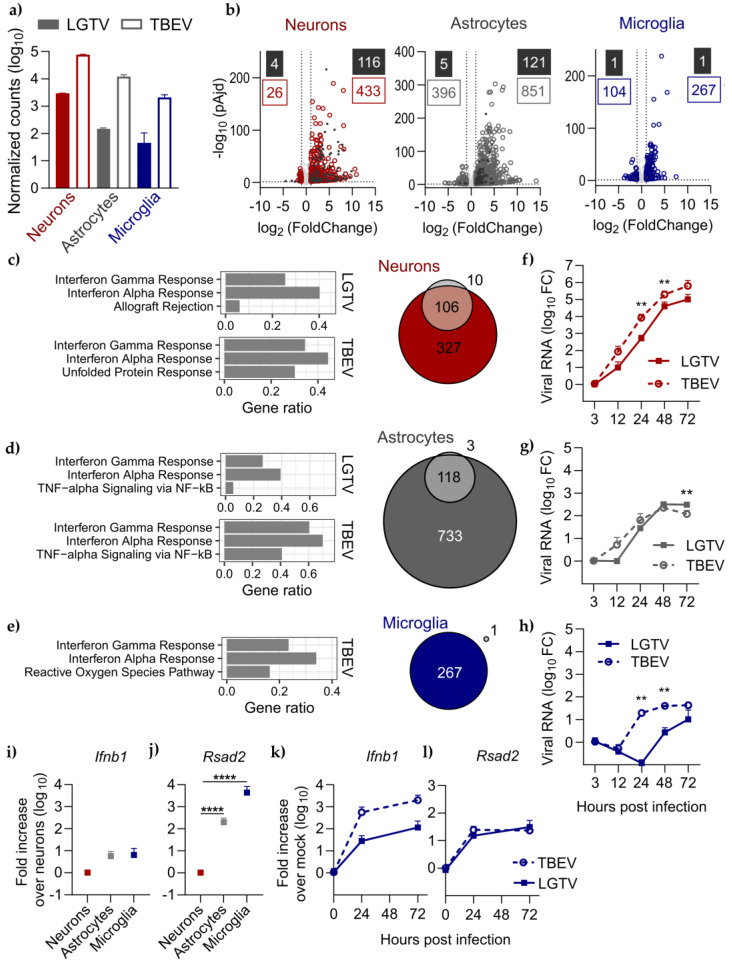
LGTV infection induces a similar but weaker immune response than TBEV in primary cells. (**a**) Infection level 24 h post-infection as normalized counts mapping to LGTV or TBEV genome in neurons (red), astrocytes (grey) or microglia (blue). (**b**) Volcano plot displaying differentially expressed genes (DEGs) upon infection in neurons (red), astrocytes (grey) or microglia (blue). Cutoff for significant genes (adjusted *p*-value < 0.05 and shrunken LFC > |1|) indicated with dotted lines. DEGs for LGTV as black squares and DEGs for TBEV as colored rings. Top 3 enriched Hallmark gene sets amongst DEGs upregulated by LGTV or TBEV infection, together with Venn diagram showing the overlap in infection-upregulated genes between LGTV (grey) and TBEV (colored) in (**c**) neurons, (**d**) astrocytes and (**e**) microglia. Viral kinetics of LGTV and TBEV in primary (**f**) neurons (MOI 0.01), (**g**) astrocytes (MOI 0.1) and (**h**) microglia (MOI 1). Viral RNA in cells quantified by qPCR and normalized to GAPDH and to the 3 h post-infection (input) using the ΔΔCt method and given as fold chnge (FC). Baseline gene expression of (**i**) *Ifnb1* and (**j**) *Rsad2* in neurons, astrocytes and microglia. Expression was normalized to GAPDH and to neurons. Gene expression of (**k**) *Ifnb1* and (**l**) *Rsad2* at indicated time points following infection with LGTV (squares and solid lines) or TBEV (circles and dotted lines) in primary microglia. Expression normalized to mock-treated cultures using the ΔΔCt method. Data from two independent experiments performed in triplicates, shown as mean and SD. Statistical significance was calculated by the Mann–Whitney test (** *p* < 0.01) (**** *p* < 0.0001).

First, we wanted to compare the transcriptional response induced by LGTV infection to TBEV infection in our primary monocultures with bulkRNA-seq. Due to differences in susceptibility between the different primary cells, we choose to infect neurons with MOI 0.01, astrocytes with MOI 0.1 and microglia with MOI 1, for 24 h. This time point was chosen to evaluate the initial response without the presence of virus-induced cell death, which occur very rapidly in the highly susceptible neurons. At this time point and MOI, not all cells in vitro are infected, similar to the situation in vivo [20]. Hence, the transcriptional response analyzed here reflects the response from both infected and bystander cells. For both viruses, we found the highest number of viral reads in neuronal cultures, followed by astrocytes and microglia (Figure 3a), even though the infectious dose was inverted. For all three cell types, we found a higher number of reads mapping to the TBEV genome than to LGTV at 24 h post-infection. The variations in gene expression profiles within the replicas from each cell type were small for both model systems (Figure S1a,b). Differential gene expression analysis of infected compared with mock-treated samples was performed using DESeq2 [30], and differentially expressed genes (DEGs) defined as log2 fold change >|1| and adjusted *p*-value < 0.05 (Figure 3b, Table S1). For LGTV, astrocytes and neurons responded to the infection with a similar number of DEGs, 120 and 126 DEGs, respectively, and a vast majority of them were upregulated. Surprisingly, only two significant DEGs (*Eml2* and *Mcu*) were identified in microglia infected with LGTV (Figure 3b). In contrast, TBEV-infected microglia showed a strong response to infection with 371 DEGs. TBEV infection induced a stronger transcriptional response than LGTV also in astrocytes and neurons, with 1247 DEGs identified in astrocytes and 459 DEGs in neurons (Figure 3b).

Next, we compared the overlap in gene signatures induced by TBEV and LGTV infection and found that almost all genes upregulated by LGTV in neurons and astrocytes were also upregulated by TBEV, 91% and 98%, respectively (Figure 3c,d). We further explored the genes induced by the respective virus by enrichment analysis using the Hallmark Gene Set curated gene lists [33]. For neurons, the top-two enriched gene sets were the same for LGTV and TBEV, namely IFN gamma and IFN alpha response (Figure 3c, Table S2). In contrast to LGTV, DEGs induced by TBEV infection in neurons were also largely enriched for untranslated protein response (UPR). Upregulated DEGs unique to TBEV in neurons included UPR-related genes *Eif2ak3* (PERK), *Atf4* and *Atf6* but not *Ern1* (IRE1) or downstream singling protein *Xbp1* (Table S1). For astrocytes, the top-three enriched gene sets were the same for LGTV and TBEV as well as IFN gamma response, IFN alpha response and TNFα signaling via NF-κB (Figure 3d). TBEV infection in microglia induced DEGs enriched for IFN gamma and IFN alpha response and reactive oxygen species pathways (Figure 3e, Table S2).

The stronger transcriptional response to TBEV compared to LGTV infection in all cell types could be due to the more rapid viral kinetics of TBEV or represent virus-specific differences in the immune response to infection. To evaluate the viral kinetic differences between LGTV and TBEV in these cells, we infected them with the respective virus and quantified viral replication over time (Figure 3f–h). This further confirmed the lower susceptibility of astrocytes and microglia compared to neurons, despite a higher MOI used (neurons; MIO 0.01, astrocytes; MOI 0.1 and microglia; MOI 1). While there was a trend of faster kinetics for TBEV in astrocytes, only microglia and neurons showed significantly higher viral replication of TBEV than LGTV at 24 h and 48 h post-infection (Figure 3f–h). In microglia, it seems that viral amounts in the cells do not increase during the first 12–24 h, especially for LGTV. This might be because microglia express high levels of restriction factors like antiviral stimulated genes (ISGs) or type I IFN at baseline level, similar to what we have shown for primary astrocytes [16]. We, therefore, compared the baseline expression of the antiviral ISG *Rsad2* (also known as Viperin) [36] and *Ifnb1* in neurons, astrocytes and microglia. We found that both astrocytes and microglia express more *Rsad2* at baseline, 200-fold and 4400-fold, respectively, than neurons (Figure 3j). This expression does not seem to be correlated with *Ifnb1* expression as no difference in basal levels was detected among the different cell types (Figure 3i). The high basal expression of *Rsad2* in microglia may contribute to the low replication at 24 h and the consequently low number of DEGs shown in LGTV-infected cells at 24 h (Figure 3e). We have previously shown that the IFN response in cell culture depends on the level of viral replication in the cells [22]. To see if the weak transcriptional response in LGTV-infected primary microglia (Figure 3b) is due to the delayed viral replication in these cells (Figure 3h), we also analyzed the expression of *Ifnb1* by qPCR at later time points. The upregulation of *Ifnb1* follows a similar trend as viral RNA, with lower levels for LGTV compared to TBEV at 24 h post-infection and a roughly 4-fold increase in levels between 24 and 72 h post-LGTV infection (Figure 3k). In contrast, *Rsad2* shows similar expression levels for both viruses at both 24 h and 72 h post-infection (Figure 3l). Together, this indicates that primary microglia are already in an antiviral state with high expression of *Rsad2,* independent of virus infection. Our data also suggest that *Rsad2* expression appears to be independent of *Ifnb1* expression in microglia. In summary, LGTV and TBEV show a similar response in vitro, albeit the response in LGTV-infected cells at the same time point is more subtle. 

### 3.3. Limited Overlap in Altered Pathways Following Infection In Vitro and In Vivo

**Figure 4:**
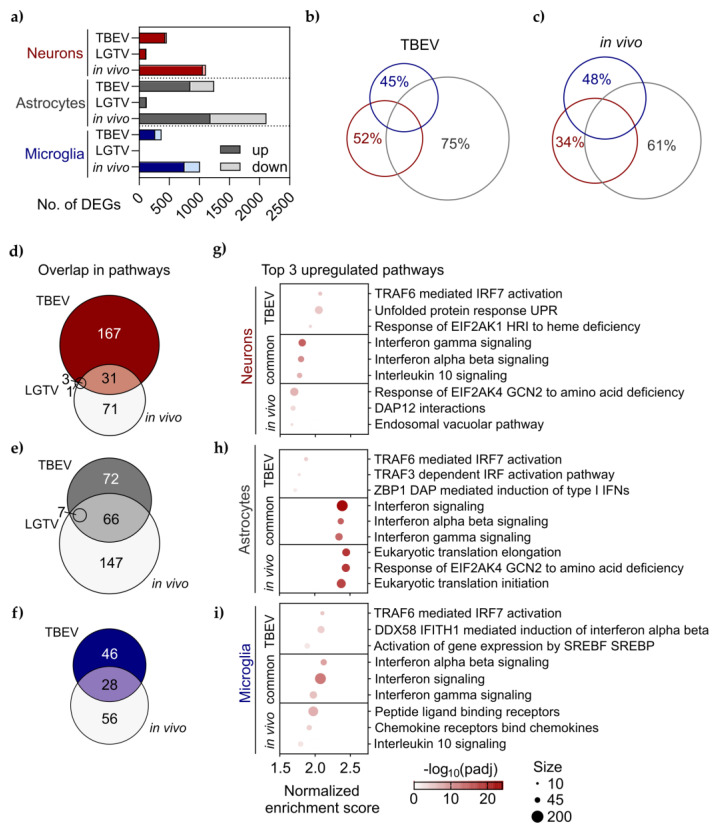
Limited overlap in altered pathways following infection in vitro and in vivo. (**a**) Number of significantly up- and downregulated genes upon infection TBEV or LGTV infection in primary cells or corresponding cell type in cerebral cortex analyzed by pseudobulk (DESeq2) of snRNAseq data (in vivo). Venn diagram showing the overlap in DEGs between cell types (neurons red, astrocytes grey and microglia blue) infected by (**b**) TBEV in vitro or (**c**) LGTV in vivo. Venn diagram showing the overlap in altered Reactome pathways following TBEV infection in vitro (colored), LGTV in vivo and LGTV infection in vivo (grey) for (**d**) neurons, (**e**) astrocytes and (**f**) microglia. Top altered Reactome pathways in (**g**) neurons, (**h**) astrocytes and (**i**) microglia. Pathways ranked based on normalized enrichment score, and top 3 unique to TBEV in vitro, shared TBEV and in vivo (common) or unique to in vivo included. The size of dots represents no. of genes mapping to that pathway and the color represents −log10 adjusted *p*-value.

Next, we compared the transcriptional response to infection of primary cells in vitro to the corresponding cell types’ response in vivo, analyzed as “pseudobulk” of snRNA seq data from a mouse cortex following LGTV infection. We observed a higher number of DEGs for all cell types in vivo compared to in vitro (Figure 4a, Table S3). There was a similar trend in responsiveness as in vitro, with astrocytes showing the highest number of DEGs (2115), followed by neurons (1107 DEGs) and microglia (1011 DEGs). We compared the overlap in DEGs between the three cell types, either as primary cells infected with TBEV (Figure 4b) or in vivo (Figure 4c). Astrocytes showed the most cell-type-specific response to infection, with 75% (TBEV) and 61% (in vivo) of all DEGs being uniquely up- or downregulated only in astrocytes, while these numbers were slightly lower for microglia (45% TBEV, 48% in vivo) and neurons (52% TBEV, 34% in vivo). The results show that in both models, a majority of DEGs were uniquely upregulated in one of the three cell types, meaning that these cell types respond differently to infection, emphasizing the importance of models enabling the study of cell-type-specific responses. Detailed knowledge of cell-type-specific responses can reveal critical host–virus interactions and drivers of pathogenesis.

Infection induced a large number of DEGs both in vivo and in vitro. To better understand the underlying biological processes that these are involved in, we performed gene set enrichment analysis using the Reactome pathway curated gene set [35]. We compared significantly altered pathways between LGTV- and TBEV-infected primary cells in vitro with cells from LGTV-infected mice in vivo. For neurons, we found only four altered pathways in vitro after LGTV infection, three of which overlapped with in vivo (Figure 4d, Table S4), and for TBEV, a higher number of altered pathways in vitro compared to in vivo (Figure 4d, Table S4). Meanwhile, astrocytes and microglia were more responsive in vivo compared to in vitro (Figure 4e,f, Table S4). Only seven significantly altered pathways were found after LGTV infection of astrocytes and they overlapped with the in vivo infection (Figure 4e). Since TBEV infection induced a strong transcriptional response in all cell types in vitro that was similar to the response induced by LGTV (Figure 3c,d,k,l), we chose to compare infection of TBEV in vitro with LGTV in vivo. A majority of the altered pathways were unique to one of the two models, suggesting important differences in response to infection in vivo and in vitro. Amongst the pathways that were commonly enriched for both conditions, interferon signaling was dominant in all cell types (Figure 4g–i). Interestingly, the top pathways unique to in vitro were TRAF6-mediated IRF7 activation for all three cell types. In vivo, the response of EIF2AK4 GCN2 to amino acid deficiency was amongst the top-three enriched pathways for both neurons and astrocytes. In addition, astrocytes in vivo showed a strong upregulation of protein translation (Figure 4h). Similar to the number of DEGs, we found the number of altered pathways to be highest in astrocytes, both in vitro and in vivo, and the common or in vivo unique pathways for astrocytes showed the highest normalized enrichment scores (Figure 4g–i).

## 4. Discussion

Primary cells of human or murine origin have been widely used as a disease model to study TBEV and other neurotropic flaviviruses [16,17,18,19,37]. While providing the advantage of studying the response by a specific cell type, it lacks information about how cells communicate and their interplay during infection. Recent advances in single-cell technology allow for detailed transcriptional profiling of single cells within a complex tissue such as the brain [38] and have been applied to study neurotropic virus infection [39,40]. However, snRNA-seq remains costly and technically demanding, especially under high biosafety containment, which may limit its widespread application. Here, we compared the transcriptional response of primary cells infected with LGTV and TBEV in vitro with the response of corresponding cell types from LGTV-infected mice in vivo. We found that comparing the in vitro and in vivo response after LGTV infection showed a strong discrepancy in responsiveness, whereas LGTV induced 101 (neurons), 213 (astrocytes) and 77 (microglia) pathways in vivo. Only four and seven pathways were significantly induced in neurons and astrocytes, respectively, in vitro, although these overlapped with the in vivo response. We also found that TBEV infection induced similar genes compared to LGTV infection in vitro; however, the response after TBEV infection was much stronger, with several hundred more genes upregulated. Comparing the response of TBEV in vitro with LGTV in vivo showed that infection in vivo only partially overlapped with the response elicited by primary cells in vitro, with overlapping pathways being dominated by the IFN response. However, we cannot fully rule out that the differences between TBEV in vitro and LGTV in vivo also to some extent represent differences induced by the viruses used. We can show that the different environments are important for how different cell types respond to infection and that care should be taken when interpreting transcriptional data from different model systems. 

We found neurons and astrocytes to be the most responsive to LGTV infection in vitro, while astrocytes followed by microglia responded strongest in vivo. We have previously shown that primary astrocytes are able to mount a rapid type I IFN-dependent response to TBEV infection [16], and others have shown that human stem-cell-derived neuronal/glial cultures mounted a stronger immune response to TBEV when astrocytes were enriched in the culture [17]. Interestingly, in our study, monocultures of microglia in vitro did not respond at all to infection by LGTV at the analyzed time point (24 h post-infection) while responding to TBEV infection at this time point and being highly responsive in vivo. This is likely a result of low infection levels due to the high basal levels of the antiviral ISGs viperin and possibly other antiviral factors that restricts infection in vitro, as no viral replication of LGTV is apparent in these cells at this time point (Figure 3h). 

Microglia are the brain’s resident macrophages and are important mediators of the immune response in the brain. As an example, microglia incorporation increased the immune response in human 3D brain spheres following ZIKV/DENV infection [41]. In mouse models, microglia are essential in protecting against neurotropic flavivirus infection, as microglia depletion increased mortality and viral titers in the brain following WNV or JEV infection [42]. At the same time, microglial activation following viral infection may trigger neuroinflammation and have long-term neurodegenerative effects if not well balanced or resolved [43]. In our previous study of LGTV infection of mouse brain, less than 4 % of microglia were infected, indicating microglial refractoriness to infection similar to the in vitro result. However, we found a strong induction of an inflammatory milieu [20]. As microglia are not the main target for LGTV infection in the brain of WT mice, microglial activation seen in vivo likely depends on exocrine signaling from other cell types, which is not seen in a monoculture system where only two genes were significantly upregulated, as measured by bulkRNA-seq 24 h post-infection. In vivo, 109 soluble ligand–receptor interactions were predicted using CellChat after LGTV infection; of these, 22 were between microglia [20]. Interestingly, even 72 h post-infection in vitro, no large changes were detected compared to the 24 h time point for *Rsad2,* as measured by qPCR.

Amongst pathways commonly upregulated in vitro and in vivo, we found IFN gamma and IFN alpha/beta signaling to be amongst the most enriched for all cell types. Type I IFNs (IFNαs and IFNβ) and their role during LGTV and TBEV infection have been well studied [16,22,25,44,45,46]. An intact type I IFN system protects mice from otherwise lethal LGTV infection and is essential both in the periphery and locally within the CNS [44]. In this study, we showed that astrocytes were the cell type showing the strongest enrichment of IFN signaling in vivo. In primary cultures, type I IFN signaling protects astrocytes against TBEV infection and the virus-induced cytopathic effect [16]. Additionally, primary astrocytes are a potent producer of type I IFNs, where their production is initially dependent on mitochondrial antiviral-signaling protein (MAVS) and later on MyD88/TRIF signaling [47]. The role of other types of IFNs during flavivirus infection has been less well studied. While the IFN gamma signaling pathway was the top enriched gene set in all cell types in vitro for both viruses, the expression of *Ifng* was not observed in our data from these cells. This is expected as IFNγ is mainly produced by T cells and NK cells, which are absent in our in vitro model system. However, IFN gamma signaling pathway appears as the top enriched gene set, possibly due to high overlap response genes with the type I IFN pathway. In contrast, cells in vivo may be exposed to IFNγ secreted by perivascular or infiltrating lymphocytes, and we recently showed that *Ifng* expressing CD8+ T cells and NK cells infiltrate mouse brains after LGTV infection [20]. In our in vivo data, we see the expression of the IFNγ receptor (*Ifngr1*, *Ifngr2*) in neurons, microglia and astrocytes, suggesting that these cells may respond to IFNγ. In line with this reasoning, we found genes encoding MHC class I to be highly upregulated by neurons in vivo (Table S1) but not at all by LGTV in vitro. MHC class I is normally expressed at very low levels in neurons; however, studies of primary neurons have shown that their expression is induced by IFNγ or other factors released from activated microglia [48]. MHC class I presents intracellular antigens to cytotoxic T lymphocytes such as CD8+ T cells, which are important in clearing virus-infected cells from CNS but also contribute to pathology during neurotropic flavivirus infection [49,50,51]. This emphasizes the fact that the crosstalk between different cell types provided in the complex environment in vivo results in a more complex transcriptional response and host defense against viral infection.

We identified TRAF6-mediated IRF7 activation amongst the top in vitro enriched pathways in all three cell types. TRAF6 belongs to the family of TNF receptor-associated factors (TRAFs) and is able to mediate the signaling from a wide range of immunoregulatory receptor families, including TLRs [52]. TRAF6 is an established part of the innate immune signaling pathways, and *Traf6*^−/−^ mouse embryonic fibroblasts (MEFs) show reduced production of, e.g., type I IFNs and IL-6 following infection with various RNA viruses [53]. Controversially, for tick-borne flaviviruses specifically, TRAF6 has been shown to instead play a proviral role through interaction with the viral protease NS3 [54]. The relative contribution of these two mechanisms in different cell types and conditions remains unclear. TRAF6 may directly activate downstream signaling via IFN regulatory factor (IRF) 7 to induce IFN production [55,56]. While IRF3 is crucial for the induction of IFNβ following TBEV infection in MEFs [22], the role of IRF7 remains less clear. Knockout of IRF7 in mice leads to a slight increase in LGTV RNA in the brain following peripheral infection but does not influence disease onset or mortality [57].

During flavivirus replication, viral proteins accumulate in the endoplasmic reticulum (ER), resulting in ER stress and the activation of UPR. Both TBEV and LGTV infections activate the UPR, but which UPR pathway (IRE1, ATF6, or PERK) is activated and its effect (pro- or antiviral) vary by cell type and virus strain [58,59,60,61]. For example, IRE1 inhibition decreased LGTV and TBEV titers in human astrocytes [58], while IRE1 silencing increased TBEV titers in U2OS cells [59]. Furthermore, PERK knockout in HEK293T cells increased LGTV titers but not those of Powassan virus [60], suggesting differences may occur between closely related viruses. In this study, we found that UPR was amongst the top enriched gene sets upregulated in astrocytes in vivo. As astrocytes are not the primary target for LGTV replication in the brain of WT mice [20], the activation of UPR in these cells is likely replication independent. Interestingly, UPR has also been suggested in priming the IFN response during flavivirus infection [59], and PERK-eIF2α signaling in astrocytes has been described as contributing to a pathogenic reactivity state of these cells during neuroinflammation [62]. We also identified UPR amongst the enriched gene sets in neurons in vitro, upregulated by TBEV but not LGTV. Gene expression suggests PERK and ATF6 as the activated pathways in these cells at this time point. In contrast, IRE1 was found to be the most activated pathway by both LGTV and TBEV infection in the neuronal cell line SH-SY5Y [58]. These differences could be due to infection conditions and kinetics or reflect a difference in cells of various origins. It should, however, be noted that thorough evaluation of UPR pathway activation requires analysis of protein phosphorylation, cleavage, and/or nuclear translocation, and, therefore, transcriptomics alone may not fully capture this phenomenon.

In addition to the environmental differences between the in vitro and in vivo models, the developmental stages of the cells used might also contribute to their distinct responses. The in vivo model comprises adult mice, while the primary cell monocultures (in vitro) are generated from pre- or post-natal mice due to the technical complexity of isolating pure cultures of live cells from older mice. The developmental stage can influence cellular physiology and immune competency, potentially affecting how cells respond to infection [63,64]. Another limitation is that we lack data on the percentage of infected cells in the primary monocultures. As a result, we cannot determine the extent to which the response originates from infected cells versus bystander cells. It is also important to consider the limitations of murine model systems to study human diseases, and similar studies for cells of human origin would be of great interest. However, there are likely similar differences between the in vitro culture models and cellular response in vivo for humans too. Since all model systems have strengths and weaknesses, care should be taken when interpreting results from different model systems.

## 5. Conclusions

The host immune response, while being essential to protect from infections, might also play a detrimental role and contribute to disease progression. In the present study, we analyzed the transcriptional response to tick-borne flavivirus infection in monocultures of primary neurons, astrocytes and microglia and compared it to the corresponding cell types’ response to infection in vivo. We found a limited overlap between in vitro and in vivo, with overlapping pathways primarily dominated by IFN signaling. Genes uniquely upregulated in vivo were related to signaling molecules not present in vitro; e.g., genes encoding MHC-I on neurons have previously been reported to be the result of factors secreted by activated microglia and were only found in vivo. Furthermore, primary microglia responded to TBEV infection but not to LGTV infection at the time point studied (24 h post-infection), probably as a result of low cellular susceptibility and slow viral kinetics in these cells. Thus, care should be taken when choosing the model system, the interpretation and the extrapolation between results from in vitro monocultures to in vivo.

## Data Availability

All computer code used for the analysis will be available upon publication at https://github.com/ERosendal/LGTV_WT_Ifnar_10x (accessed on 14 August 2024). Raw bulk RNA-seq data are available at ArrayExpress #E-MTAB-12902.

## Supplementary Materials

The following supporting information can be downloaded at: https://www.mdpi.com/article/10.3390/v16081327/s1, Figure S1. Principal Component Analysis (PCA) of transcriptomic data showing variation between replicates (**a**) in vitro and (**b**) in vivo, colored by cell type and treatment. The percentage of the total variance explained by principal components 1 and 2 is indicated on respective axis. Table S1: Differentially expressed genes in primary neurons, astrocytes and microglia infected with LGTV or TBEV in vitro compared to mock; Table S2: Enriched Hallmark gene sets amongst differentially expressed genes in primary neurons, astrocytes and microglia infected with LGTV or TBEV in vitro compared to mock; Table S3: Differentially expressed genes in neurons, astrocytes and microglia in vivo after LGTV infection compared to mock; Table S4: Significantly altered Reactome pathways in neurons, astrocytes and microglia, either infected with LGTV in vitro (only neurons and astrocytes), TBEV in vitro or from LGTV-infected mice in vivo, compared to respective mock.
