# Supplementary figures and images for: Transcriptional Response to Tick-Borne Flavivirus Infection in Neurons, Astrocytes and Microglia In Vivo and In Vitro
Source: Viruses. 2024 Aug 19;16(8):1327. doi: 10.3390/v16081327 (PMC11359927; doi:10.3390/v16081327)

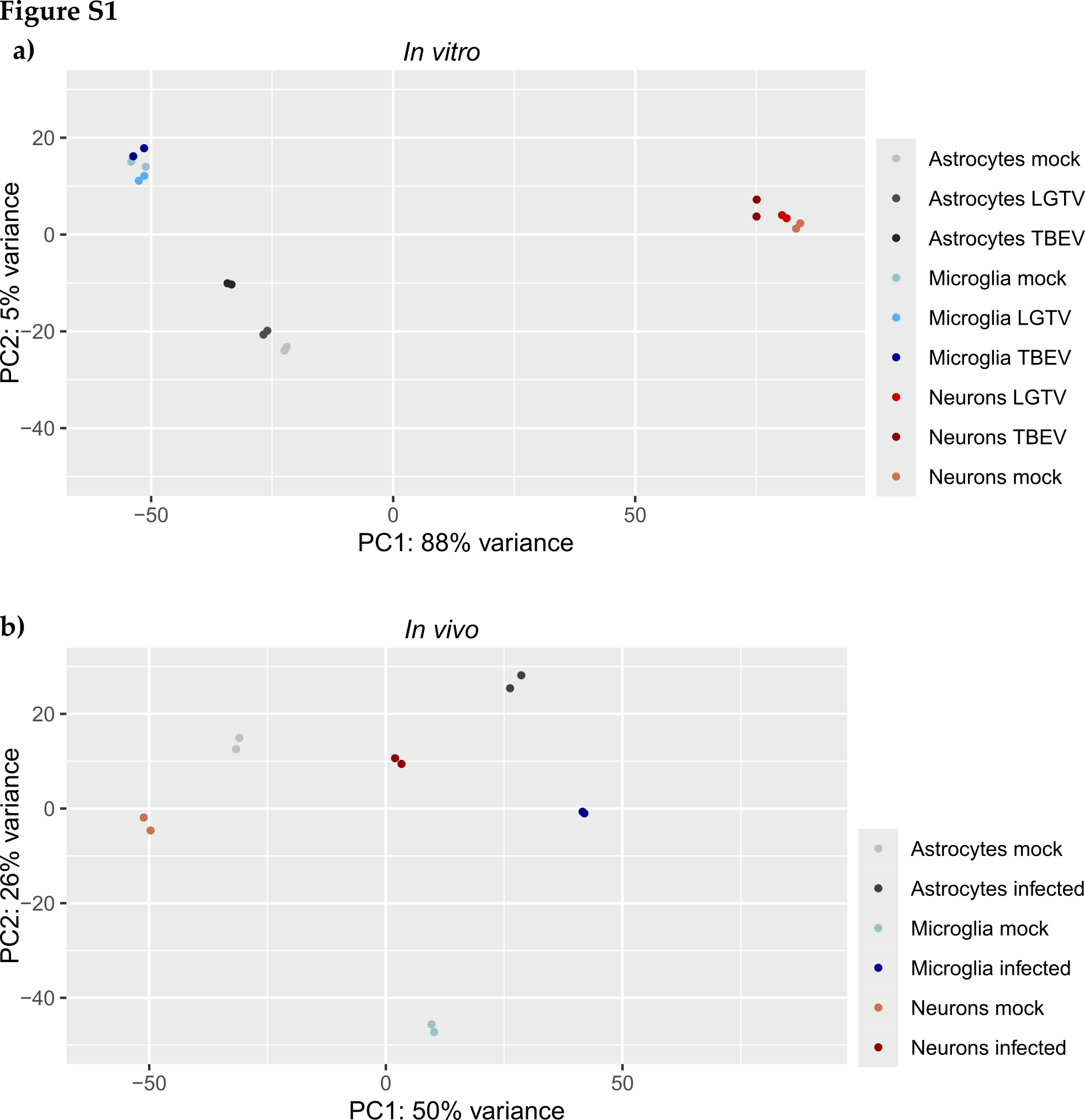

Supplement: Supplementary file 1 [file viruses-16-01327-s001.zip › FigureS1_updated.png]
